# Shaping the Space: A Role for the Hippocampus in Mental Imagery Formation

**DOI:** 10.3390/vision9010002

**Published:** 2025-01-08

**Authors:** Andrea Blomkvist

**Affiliations:** Centre for the Study of Perceptual Experience, Department of Philosophy, University of Glasgow, Glasgow G12 8QQ, UK; andrea.blomkvist@glasgow.ac.uk

**Keywords:** mental imagery, hippocampus, episodic memory, spatial model, scene construction theory, aphantasia

## Abstract

Mental imagery is claimed to underlie a host of abilities, such as episodic memory, working memory, and decision-making. A popular view holds that mental imagery relies on the perceptual system and that it can be said to be ‘vision in reverse’. Whereas vision exploits the bottom-up neural pathways of the visual system, mental imagery exploits the top-down neural pathways. But the contribution of some other neural areas remains overlooked. In this article, I explore important contributions of the hippocampus, a neural area traditionally associated with episodic memory, to mental imagery formation. I highlight evidence which supports the view that the hippocampus contributes to the spatial model used for mental imagery and argue that we can distinguish different hippocampal circuits which contribute to different kinds of imagery, such as object imagery, scene imagery, and imagery with a temporal aspect. This has significant upshots for mental imagery research, as it opens a new avenue for further research into the role of the hippocampus in a variety of imagery tasks.

## 1. Introduction

Try to remember your graduation. For many of us, remembering this will evoke a mental image. We visualise what the remembered scene looked like, including details of who was there, what they were wearing, where they were standing, and so on. These mental images can aid memory. If asked about details of the scene, I can mentally scan the scene to find the answer. In short, we evoke a mental image of the scene, and this mental image supports our ability to recall details from the event [1]. Similarly, we can evoke a mental image of a future event which we have not yet experienced, though this tends to be less vivid and not as detailed as images of the past [2].

Notably, mental images are not only generated in the visual modality but exist in a host of modalities including audition, taste, smell, and emotion (see Andrade et al. [3] for a subjective measure of different kinds of imagery). ‘Mental imagery’ is sometimes used as an overarching term for all kinds of imagery and sometimes more narrowly to refer to ‘visual imagery’ only. In this article, I use it in the more narrow sense, unless otherwise stated. Moreover, though commonly associated with memory and imagination, the generation of an image is not necessary for any of these experiences, as demonstrated by research into aphantasia—an individual difference in the generation of voluntary visual imagery, which is associated with reduced (but not absent) detailedness in autobiographical memory and future imagination [4,5,6].

In what follows, I am interested in cases where we do generate a visual image when we imagine the future or recall the past. What neural mechanisms contribute to the generation of this image? Research has long supported the involvement of the visual system in visual imagery generation, where it has been suggested that generating a visual image is like ‘vision in reverse’ [7]. When generating visual imagery, the suggestion is that the visual system is driven in a top-down way by the prefrontal cortex and that content from memory is recruited to then be processed in a reverse hierarchy in the visual system, from high-level areas to low-level areas. Visual imagery and perception are hence thought to share many of the same neural mechanisms [8].

However, mental imagery research has mostly focused on the role of the visual cortex in mental imagery formation, whereas other neural areas which are potentially implicated in the process are relatively underexplored and little is known about their function. One such area is the hippocampus, traditionally thought to support episodic memory. Though it is often claimed that mental imagery takes its content from episodic memory, it has been pointed out that the role of the hippocampus in mental imagery formation is particularly unclear, and some research might even suggest that its involvement is not necessary [7].

In this article, I aim to clarify the role of the hippocampus in mental imagery formation in humans. Though previous work has focused on the role of the hippocampus in imagination, this work has not discussed the contribution to mental imagery formation specifically. The distinction between imagination and mental imagery is an important one which has long been recognised in philosophy, whereby a mental act of imagining is not necessarily accompanied by mental imagery (see Box 1). I aim to provide a novel insight into the role of the hippocampus for mental imagery formation by drawing on work in neuroscience. I argue that we can already discern different hippocampal circuits involved in generating different kinds of imagery, such as object imagery and scene imagery, and that the hippocampus likely supports the building of a spatial model for mental imagery. This has an upshot for the study of mental imagery as it implies that different tasks put different demands on the hippocampus, just as how different tasks put different demands on various visual areas. I argue that this insight is especially important for the study of special populations.

In Section 2, I first give a brief overview of neural areas which are broadly taken to be associated with the generation of mental imagery, before homing in on the hippocampus. I argue that the hippocampus contributes to the formation of both object imagery and scene imagery by providing a spatial model. This is corroborated by neuroscientific evidence showing different activation patterns, as well as data showing deficits in the spatial coherence of mental imagery in a clinical population. I further argue that the hippocampus could play different roles depending on the temporal direction of imagery. In Section 3, I put forward evidence suggesting that the hippocampus is not involved in mental imagery formation, and I suggest that this conclusion does not follow as other plausible interpretations of the data are available. In Section 4, I discuss the consequences for mental imagery research and suggest that researchers ought to be sensitive to what tasks are set, especially when studying special populations, as hippocampal circuits supporting different kinds of imagery could potentially dissociate. Finally, in Section 5, I conclude that we ought to further investigate the role of the hippocampus in imagery formation, as there are many aspects left to explore.

Box 1 **Propositional imagination and sensory imagination.**

Philosophers make a distinction between propositional imagination and sensory imagination [9]. Propositional imagination involves imagining that something is the case (e.g., imagining that *Paris is not the capital of France*) whereas sensory imagination involves deploying one’s sensorimotor systems to evoke a mental image (e.g., imagining *walking down the street of Paris and hearing the sound of French being spoken*). Mental imagery is taken by many to be a necessary component of sensory imagination, but not of propositional imagination.

## 2. The Mechanisms of Mental Imagery Generation

The generation of mental imagery is a complex process associated with multiple neural areas. To better contextualise the contributions of the hippocampus, I briefly discuss the contributions of other areas, including prefrontal areas and visual areas (for a detailed discussion, see [7,10]). I also limit my discussion here to the formation of voluntary visual imagery, as this is what most research focuses on.

### 2.1. Visual Cortex and Prefrontal Areas

The investigation of the neural processes of visual imagery has a long history. Most research has focused on the relationship between visual perception and visual imagery, asking to what extent visual areas support the creation of visual imagery. It has been hypothesised that the creation of visual imagery is like ‘vision in reverse’ [7], a reverse hierarchy where prefrontal areas drive activation in visual cortices, from higher to lower levels (for a recent discussion, see Dijkstra [11]). Particular attention has been paid to the potential activation of V1, which has been hypothesised to be necessary for the generation of visual imagery ([10] but see also [12,13]), and wherefrom researchers have been able to decode the content of mental imagery [14]. This suggests that there are common patterns of activation for perception and visual imagery, which become increasingly similar at higher cortical levels but which can be detected as early as V1. The prefrontal cortex (PFC) is also highlighted as an area which contributes to mental imagery formation. As this area is active in a wide range of imagery tasks, it is likely that it plays a more executive role in driving the activation of subsequent areas [15,16,17]. It is also possible that it selects schema on which imagery is scaffolded [18,19].

### 2.2. Anatomy of the Hippocampus

Before discussing the contributions of the hippocampus to mental imagery formation, it is useful to know about the functional neuroanatomy of the hippocamps. The hippocampus is part of the default mode network (DMN), which is involved in both remembering and imagining scenes and events [20,21]. This network comprises the hippocampal formation (hippocampus, enthorinal cortex, subiculum, presubiculum, parasubiculum and the parahippocampal gyrus [22]. The hippocampus itself can be divided into three parts—head, body, and tail. The body of the hippocampus is divided into cytoarchitectonically defined subfields comprising the dentate gyrus (DG), CA3, CA2, and CA1. These form a closed circuit where information is inputted via the enthorinal cortex, which in turn drives DG, CA3, CA2, and CA1 and the subiculum. The subiculum and CA1 then project back to the EC and the loop is closed. Interestingly, recent research has also demonstrated direct connections between the hippocampus and various cortical areas using in vivo quantitative fibre tracking in humans (for similar work on primates, see [23]). This work has demonstrated preferential connectivity along the anterior-posterior hippocampal axis—for example, between the tail of the hippocampus and occipital areas such as V1 and V2. Areas in the occipital cortex also display higher endpoint density in the posterior medial hippocampus and, to a lesser degree, in the anterior medial hippocampus. These anatomical links could be crucial for mental imagery formation, as discussed later.

In terms of function, the hippocampus has long been an area which we know to be important for episodic memory encoding and retrieval, but research has also shown that it is involved in future episodic thought [20,21], counterfactual thinking [24], navigation [25], as well as perception [26,27,28]. Research on non-human animals has also contributed greatly to our understanding of the function of the hippocampus, potentially supporting its playing a role in mental imagery generation. For example, research has shown that the dorsal hippocampus in rats plays a role in filling in missing information, potentially as a visual image, of visual cues (for an overview, see [29]). Techniques using a machine interface have also supported the volitional access of cognitive maps to navigate a virtual reality environment in rats, a strategy which is akin to imagining a possible route [30]. However, as pointed out [31], as there are anatomical differences between the human and non-human hippocampus, we need to be careful when generalising about function from one species to another. For the remainder of this article, I limit my discussion to the role hippocampus plays in imagery formation in humans, especially as I want to focus on voluntary imagery, something which is difficult to assess in other animals.

This new research in both human and non-human animals puts pressure on the old idea that the hippocampus is the ‘seat of memory’ and that its sole function is to support episodic memory. Instead, another suggestion is that the hippocampus is involved in the more general processing of scenes, whether these are perceived, imagined, or remembered [26,32]. Scene construction theory suggests that the hippocampus is responsible for constructing the spatial representation necessary for creating a spatially coherent scene. Supporting research shows that hippocampal lesion leads to an inability to construct spatially coherent scenes [33], and fMRI studies support the involvement of the hippocampus especially in tasks which require a 3D spatial layout [29,30,34,35,36].

But how does this complex system contribute to mental imagery formation? So far, the emphasis in research has been on how the hippocampus, as part of the DMN, contributes to episodic recall or future imagination by supplying content of past experiences. However, neither of these functions are necessarily accompanied by mental imagery, as we can propositionally imagine the future or remember the past (see Box 1), and many studies do not assess whether participants engage in mental imagery, or whether they merely think about a past or future event without having an accompanied experience of imagery. Luckily, a handful of studies have assessed whether participants engage in mental imagery formation during these tasks by using subjective measures such as vividness ratings. I highlight these studies as informative regarding the role of the hippocampus in mental imagery formation. I argue that, in line with scene construction theory, data suggest that the hippocampus plays the role of building a spatial representation in mental imagery during scene imagery and object imagery. Scene imagery involves visualising a 3D space populated with objects, which one could potentially ‘step into’, such as a jungle or a living room. Object imagery involves visualising one or multiple objects, but where these are located on a plain background rather than in a wider scene. Further, the hippocampus also contributes to the temporal direction represented in mental imagery [37]. Interestingly, as we will see, the role of the hippocampus in different kinds of imagery formation is nuanced.

### 2.3. Hippocampal Contributions to Object Imagery vs. Scene Imagery

An fMRI study by Dalton et al. [34] investigated the contribution of the hippocampus to scene imagery vs. object imagery. This experiment involved scanning participants whilst they were asked to visualise the following: an object, a 2D grid, arrays of objects placed on a 2D grid, a 3D grid, and objects placed on a 3D grid (referred to as ‘scene imagery’). All cases were instructed to be static images, where perspective was not shifted. For all tasks, the participants focused on a white screen with their eyes open and were explicitly told not to recall from memory. For imagining objects, the participants were presented with three separate object descriptions and asked to imagine these objects on a plain background one at a time. For a 2D grid, they were simply asked to imagine a 2D grid covering two-thirds of the white screen. For the array of objects, they were asked to imagine the 2D grid and then ‘place’ three objects on it at separate locations. For the 3D grid, they were asked to imagine a 3D grid stretching out over two-thirds of the white screen. And finally, for scene imagery, they were first asked to imagine the 3D grid and then ‘place’ three separate objects in it at different locations. Crucially, the participants rated their vividness of each imagined item on a scale between 1 and 5 (1 being ‘not at all’, and 5 being ‘extremely vivid’), and only cases which were rated as 4 or above were included in the analysis. As Dalton et al. were interested in testing the contributions of the hippocampus in imagining scenes, they were primarily interested in contrasting the cases of imagining an array of objects on a 2D grid with scene imagery. However, for my present purposes, further contrast cases are also of interest, as they are telling in how the hippocampus could potentially contribute in different ways to imagery formation.

The fMRI results showed that the hippocampus, as well as other brain areas, were differentially activated depending on the task. That is, depending on what kind of imagery a participant formed, different subregions of the hippocampus were active. First, contrasting the imagery of objects with scenes revealed bilateral activation in the perirhinal cortex (PRC), entorhinal cortex (ENT), and right anterior lateral hippocampus. Contrasting scene imagery with the imagery of objects instead showed activation in the bilaterial pre/parasubiculum, parahippocampal cortex (PHC), retrosplenial cortex (RSC), and posterior cingulate cortex (PCC). Second, contrasting imagining an array of objects with constructing a scene engaged the bilateral ENT, PRC, early visual cortices (EVC), and left posterior hippocampus. This is consistent with previous studies which have also shown greater activation in the occipital cortex during object imagery [36]. Contrasting constructing a scene with imagining an array of objects instead showed activation in the anterior medial hippocampus, PHC, RSC, and PCC.

The activation of these different areas is in line with previous findings. These studies have shown that portions of the pre/parasubibulum in the anterior medial hippocampus are involved in scene construction [38], and Dalton et al. suggest that these areas support the construction of scenes couched in a naturalistic 3D spatial framework. This is particularly plausible as this area, due to its anatomical location [39], could have access to holistic representations of the environment and could be a ‘hub’ for scene-based recognition [38]. Evidence from connectivity studies [40] showing that the hippocampus displays greater connectivity to visual areas than previously thought further bolsters this possibility. Based on these results, Dalton et al. concluded that the hippocampus (as well as other areas) is preferentially activated depending on the task and that there are multiple processing circuits within the hippocampus which engage depending on the task demand.

A further study which investigated the temporal dynamics of scene imagery and object imagery using MEG similarly supports the claim that different circuits are involved [17]. The participants were asked to either imagine a 3D space that they could step into (such as a jungle) (there were no instructions regarding whether this should be a static scene or a temporally unfolding scene, so it is possible that the participants could have imagined it as temporally unfolding), imagine a single object on a white background (such as a cushion), or perform a baseline task (counting backwards). They were explicitly told not to recall from memory. The results showed that the left anterior hippocampus showed a significant change from baseline in terms of a theta power decrease in both imagery tasks. These results corroborate Dalton et al.’s (2018) findings that the anterior hippocampus is involved in scene imagery [34,41]. Interestingly, there was also an observed theta increase between vmPFC and the hippocampus, which was larger when imagining scenes than when imagining objects. Barry et al. suggest that this could be due to vmPFC being involved in extracting regularities across scenes to form schemas, which can then serve as ‘templates’ for novel scenes [18,19]. Hence, here too, we see that the hippocampus (in conjunction with vmPFC) is involved in both scene imagery and object imagery, but these two tasks are supported by different circuits (for a broader discussion on scene and object processing within the hippocampus, see [42]).

Finally, a telling study suggests that lesions to the hippocampus also have consequences for the mental imagery formation of scenes, where a phenomenon known as ‘boundary extension’ is not experienced by patients with bilateral hippocampal lesion [33]. Boundary extension occurs when we recall parts of a scene which were beyond our actual viewpoint at the time [43], and whether it occurs is now thought to depend on the properties of the stimuli [44]. Unlike the controls, the patients did not experience this effect in a number of paradigms which required them to either draw or report on a previously viewed photograph. When asked what could be beyond the scene that they had previously viewed in the photograph, they were able to recount objects that could be present but unable to say how these would spatially relate to the scene they had seen. These results strongly suggest that the hippocampus is involved in building a coherent spatial representation of a scene and that lesion to the hippocampus impairs this ability.

### 2.4. Hippocampal Contributions to Mental Imagery with a Temporal Aspect

Mental imagery can also unfold over time and hence contain a temporal aspect. That is to say, some mental imagery can be more like a mental video, where subjects do not just experience one snapshot but rather the unfolding of an event. Some studies have investigated the role of the hippocampus in mental imagery of past and future events which contain this temporal aspect. For example, Addis et al. [37] investigated participants who were tasked with constructing a past event or a future event and contrasted a construction phase of the event with an elaboration phase. A conjunction analysis comparing the imagery of a past and future event to a control task revealed that a number of regions were commonly recruited, including the left hippocampus, right inferior parietal lobule, left superior occipital gyrus/cuneus, and right middle occipital gyrus. However, comparing the construction phase to the elaboration phase did not reveal any significant differences in hippocampal activations but did elicit differences in other brain areas. Hence, the hippocampus might contribute to a similar extent to both the construction phase and the elaboration phase. It is also possible here that different sub-regions of the hippocampus contribute differently, similar to the findings previously discussed, but sub-regions of the hippocampus were not assessed separately in this study. Notably, another temporal aspect that mattered to the areas activated was temporal direction, that is, whether the event was represented as being in the past or the future. For both temporal directions, the left hippocampus was equally activated. However, comparing the imagery of a future event with the imagery of a past event, the right hippocampus was significantly more activated during the creation of future events. This suggest that the temporal direction represented in imagery also elicits different responses for the hippocampus and that it could serve a role in generating non-imagistic content in mental imagery, such as tagging an event as representing the past or future (for a similar idea regarding the format of mental imagery as being ‘cartographic, see [45]).

### 2.5. Summary

What does this tell us about mental imagery formation? As different hippocampal circuits are involved depending on task demand, this suggest that different subregions of the hippocampus (and surrounding areas) make different contributions to various kinds of imagery. Interestingly, this mimics findings in vision science where different areas of the visual cortex are involved in the formation of different kinds of imagery, such as areas associated with object recognition being active for object imagery [10]. However, we have not known that similar patterns due to task demand can be traced in the hippocampus. Scene construction theory could explain these findings by appealing to a more complex spatial layout being required for constructing a 3D scene, compared to constructing an object in a 2D space. Potentially, different circuits support the construction of 3D space vs. 2D space, and hence, we see one circuit being recruited when participants are asked to construct scene imagery and another circuit being recruited when participants are asked to construct object imagery. Another possibility is that the different circuits reflect the complexity of the task, where scene imagery is generally more complex than object imagery as it often involves more features and more complex relations between objects. Moreover, the temporal direction also elicits different responses from the hippocampus, suggesting that the hippocampus could play a role when it comes to representing imagery with different temporal content. As such, it might be more apt to say that the hippocampus provides a spatiotemporal model for imagery formation, rather than only a spatial model.

To sum up, even though few studies have been conducted which specifically investigate the contributions of the hippocampus to mental imagery formation, we can already detect a pattern whereby different subregions of the hippocampus and related extra-hippocampal areas support different imagery tasks. Now, though these studies demonstrate a role for different parts of the hippocampus in the generation of different imagery representations, speculating about the function of these different anatomical parts would be premature, as the evidence so far is fairly scarce, making it difficult to form specific testable hypotheses. Future research should extend these findings—for example, by looking at what demands are placed on the hippocampus when engaging in even more complex imagery formation, such as imagery formation in different modalities (e.g., visual, auditory, emotion) or imagery of future plausible/implausible events [46]. Another possible function of the hippocampus in mental imagery generation could be sampling from a generative model during both perception and imagery [47], where more such sampling would be required for generating a scene compared to a single object. This proposal appears compatible with the data presented in this article, as it posits a neurocomputational model explaining how the hippocampus could achieve the higher-level function of providing a spatiotemporal model. Note that my claims in this article are not about how the function is accomplished on a neurocomputational level. Future research should also further investigate this hypothesis.

## 3. Evidence Against Hippocampal Involvement in Mental Imagery Generation

Before moving on, I will address two findings which purportedly go against my suggestion in this article. First, Maguire et al. [48] conducted a study which was subsequently reported as failing to find a role for the hippocampus in imagery formation (Pearson, 2019). Maguire et al. report on two amnesic patients (one with acquired amnesia and one with developmental amnesia) and their ability to imagine fictitious events and future experiences. Interestingly, both these patients were able to construct these scenarios to the same levels of detailedness and spatial coherence as controls. This is surprising if the hippocampus is crucial for imagery generation, and especially if it is crucial for generating 3D spatial scenes. Hence, it might be tempting to say that this study shows that the hippocampus is not crucial for scene imagery generation. However, there is a possible explanation for these patients’ intact ability, as 50% of the patients’ hippocampi remained intact and showed activation on MRI. It is hence possible that they could perform these tasks with support from the residual hippocampi tissue, as suggested by Maguire et al. in the original study. Note that vividness was not explicitly rated in this study; participants instead evaluated *saliency* from ‘could not see anything at all’ to ‘extremely salient’, which uses very similar terminology to a standard vividness questionnaire.

Second, another study which could cast doubt on the role of the hippocampus in mental imagery formation [7] tested the ability of amnesic patients with bilateral hippocampal damage regarding spatial transformation tasks [49]. These included mental rotation, imagery for spatial location, mental paper folding, and the Mental Imagery Questionnaire. They found no differences in performance between patients and controls and concluded that the hippocampus is not necessary for forming the mental imagery required for solving these tasks. This seems surprising, as solving these tasks is thought to depend on creating mental imagery with complex spatial relations (e.g., in the mental rotation task). However, other research shows that these tasks can be solved without appealing to mental imagery, as alternative strategies are available. This interpretation is supported by a recent study of participants with aphantasia (defined here as having reduced or no imagery ability) [50], which found that these participants gave more accurate but slower responses to mental rotation tasks. Interestingly, accurate but slower response time is also what Kim et al. reported when testing amnesiac patients. What sheds further light on the issue is that Kay et al. additionally queried participants on what strategy they used to solve the task. This revealed that aphantasics had a preference for analytic, rather than rotation-based, strategies—they were most likely not solving the task by using mental imagery [51].

Hence, mental rotation tasks can be solved without the use of mental imagery, and it is possible that patients in Kim et al.’s study used alternative strategies to solve the task. For the remaining tasks tested by Kim et al., similar possible strategies for solving the tasks were not investigated or ruled out, and it remains possible that these too could be solved by alternative strategies as it is not clear that mental imagery is necessary for any one of them (even if many people may use mental imagery as a strategy). In fact, similar results have been found in other working memory tasks [51]. To conclude, there is no clear evidence against the suggestions that the hippocampus contributes a spatiotemporal model to mental imagery.

## 4. Upshot for Mental Imagery Research

The involvement of the hippocampus in mental imagery formation is consequential for how we should design tasks and questionnaires to investigate mental imagery. Mental imagery questionnaires are frequently used in neuroscience and psychology to assess mental imagery ability, such as in aphantasia research (for a recent discussion, see [52]) or across a lifespan [53]. Of particular note are two widely used questionnaires: the VVIQ [54,55] and the Plymouth Sensory Imagery Questionnaire (PSI-Q) [3]. These questionnaires comprise questions which elicit scene imagery and object imagery, and some of these questions ask participants to explicitly draw on memory. For example, half of the questions (8/16) of VVIQ explicitly focus on eliciting imagery based on past memory, including a relative or friend that one knows well, and the front of a shop which one frequently visits. The other questions are more generic (a sunset, and a country scene) and do not ask subjects to draw on past memory (though subjects could of course still do so). In contrast, PSI-Q contains 35 questions, of which 5 focus on visual imagery, and 2 of these could be thought to cue memories (“imagine a friend you know well” and “imagine the appearance of the front door of your house”), whereas the others are more generic (e.g., a cat climbing a tree, a bonfire). No questions in non-visual modalities explicitly call on subjects to use their memory. It is also interesting to note that some questions ask participants to imagine a scene (e.g., a country scene from the VVIQ, or a cat climbing a tree from the PSI-Q), whereas others ask them to imagine single objects (e.g., a bonfire from the PSI-Q, or the door of the shopfront from the VVIQ).

Based on the previous discussion, we should expect differential contributions of the hippocampus to these heterogeneous tasks, as some involve explicitly drawing on memory, some involve object imagery, some involve scene imagery, and some also involve different temporal aspects. Though these kinds of questionnaires might give us a good overview of someone’s ability to generate mental imagery by testing heterogeneous tasks, they could also potentially obscure differences. For example, if assessing clinical populations (especially populations with hippocampal lesions), we need to pay close attention to what kind of imagery we are asking a patient to form, as it could be the case that a deficit only encompasses one kind of imagery (e.g., scene imagery) even if another kind of imagery (e.g., object imagery) could be formed.

This is not to say that mental imagery should only be assessed by subjective questionnaires, as this method alone cannot tell us about the neural mechanisms of mental imagery formation, and there is also the added concern of subjects being required to introspect their mental state, which could be unreliable [56]. Philosophers have further pointed out that central concepts used in mental imagery research—notably ‘vividness’—are poorly understood and not appropriately defined in questionnaires [57]. Tackling these questions is central for mental imagery research to move forward, and interdisciplinary research projects would be an ideal way of making headway here, as skills from philosophy, neuroscience, and psychology are required.

Moreover, paying closer attention to the kind of imagery we ask participants in experiments to form could also reveal new findings in non-clinical populations, such as people with aphantasia. We already have reason to believe that people we currently classify as aphantasic actually make up a heterogeneous group [52], as there are varying reports from people who cannot form imagery in any modality, people who cannot form visual imagery but can form non-visual imagery, and people who can form involuntary but not voluntary visual imagery [58]. Different underlying neural mechanisms could give rise to these different symptoms [59]. Moreover, recent research also points to different hippocampal activity in the aphantasic population compared to controls when retrieving autobiographical memories [6]. As this indicates hippocampal involvement, it warrants asking more nuanced questions as to what aspects of content in imagery people with aphantasia could potentially form. As aphantasia is most commonly defined as reduced, rather than absent, voluntary visual imagery, studies include participants reporting ‘vague and dim’ imagery in the aphantasic category. However, which aspects of imagery this subgroup (sometimes referred to as ‘hypophantasic’ [60]) can form has not been investigated. A possible aspect which should be investigated is whether there are differences when it comes to scene imagery, object imagery, and imagery based on the temporal direction between this group and controls. More nuanced thinking about the role of the hippocampus in mental imagery formation should lead us to hypothesise that there could be hitherto unnoticed differences in responses to imagery tasks as a result of the contributions of different hippocampal circuits.

## 5. Conclusions

I have argued based on neuroscientific evidence that it is likely that the hippocampus plays a role in mental imagery formation by building the spatiotemporal model. Though we still do not know exactly which function different sub-regions perform, evidence suggests that the hippocampus does instantiate different functions depending on task demand, where different circuits are deployed depending on what kind of imagery is formed. This has significant upshots for mental imagery research, which up until now has mostly focused on the role of the visual system in imagery formation. We should further investigate the role that the hippocampus plays, and researchers ought to be sensitive to the hippocampus’ differential activation patterns when designing tasks and questionnaires. This is of particular importance when studying special populations, as this could potentially reveal more about the involvement of hippocampal subregions in forming mental imagery.

## Data Availability

No new data were created or analysed in this study. Data sharing is not applicable to this article.
